# Can Birds Perceive Rhythmic Patterns? A Review and Experiments on a Songbird and a Parrot Species

**DOI:** 10.3389/fpsyg.2016.00730

**Published:** 2016-05-19

**Authors:** Carel ten Cate, Michelle Spierings, Jeroen Hubert, Henkjan Honing

**Affiliations:** ^1^Behavioural Biology, Institute of Biology Leiden and Leiden Institute for Brain and Cognition, Leiden UniversityLeiden, Netherlands; ^2^Amsterdam Brain and Cognition, Institute for Logic Language and Computation, University of AmsterdamAmsterdam, Netherlands

**Keywords:** rhythm perception, songbirds, parrots, perceptual bias, local vs. global information

## Abstract

While humans can easily entrain their behavior with the beat in music, this ability is rare among animals. Yet, comparative studies in non-human species are needed if we want to understand how and why this ability evolved. Entrainment requires two abilities: (1) recognizing the regularity in the auditory stimulus and (2) the ability to adjust the own motor output to the perceived pattern. It has been suggested that beat perception and entrainment are linked to the ability for vocal learning. The presence of some bird species showing beat induction, and also the existence of vocal learning as well as vocal non-learning bird taxa, make them relevant models for comparative research on rhythm perception and its link to vocal learning. Also, some bird vocalizations show strong regularity in rhythmic structure, suggesting that birds might perceive rhythmic structures. In this paper we review the available experimental evidence for the perception of regularity and rhythms by birds, like the ability to distinguish regular from irregular stimuli over tempo transformations and report data from new experiments. While some species show a limited ability to detect regularity, most evidence suggests that birds attend primarily to absolute and not relative timing of patterns and to local features of stimuli. We conclude that, apart from some large parrot species, there is limited evidence for beat and regularity perception among birds and that the link to vocal learning is unclear. We next report the new experiments in which zebra finches and budgerigars (both vocal learners) were first trained to distinguish a regular from an irregular pattern of beats and then tested on various tempo transformations of these stimuli. The results showed that both species reduced the discrimination after tempo transformations. This suggests that, as was found in earlier studies, they attended mainly to local temporal features of the stimuli, and not to their overall regularity. However, some individuals of both species showed an additional sensitivity to the more global pattern if some local features were left unchanged. Altogether our study indicates both between and within species variation, in which birds attend to a mixture of local and to global rhythmic features.

## Introduction

In 1871, Darwin wrote: “*The perception, if not the enjoyment, of musical cadences and of rhythm is probably common to all animals and no doubt depends on the common physiological nature of their nervous systems”* (Darwin, 1871). At the time, this thought was understandable as many animal species show behaviors that are characterized by some form of rhythmicity. It can be found in invertebrates, like the flashing patterns of fireflies, which can even be synchronized (Buck, 1988), as well as in vertebrates, like the strong rhythmicity characterizing some bird vocalizations. For instance, the cooing of the collared dove (*Streptopelia decaocto*) consists of a series of repeated “coos,” each consisting of three vocal elements of different duration separated by brief pauses, also of unequal duration. This temporal pattern, and hence the structure of the coo as a unit, is highly stereotyped (Ballintijn and ten Cate, 1999), resulting in a characteristic rhythmic pattern for a series of coos. Receivers are very sensitive to the overall regularity of the coo: if the temporal structure is changed, the responses are strongly reduced (Slabbekoorn and ten Cate, 1999). The question is whether, as Darwin implied, such examples indicate a sensitivity to rhythmicity in general (ranging from a sensitivity for rhythmic pattern, pulse, and meter, as well as the influence of tempo and timing; Honing, 2013) or whether this sensitivity is confined to particular species specific behaviors. Below, we will first review this topic, with particular attention to the “vocal learning and rhythmic entrainment hypothesis” formulated by Patel et al. (Patel, 2006; Schachner et al., 2009; Patel et al., 2009a,b). Doing so, we focus on studies on the perception of rhythmic patterns in birds, which for various reasons provide an ideal group for comparative studies on this topic. Next we present experimental data on pattern perception and the responsiveness to tempo changes in a songbird (zebra finch) and a parrot species (budgerigar).

### The vocal learning and rhythmic entrainment hypothesis

The interest in rhythm perception in animals is part of the more general quest for searching for signs of musicality in non-human animals, as a means to get more insight in the evolutionary and causal processes underlying human musicality (Hoeschele et al., 2015; Honing et al., 2015). The specific question whether animals can detect regularity in a stimulus and synchronize their own behavior to arbitrary rhythmic patterns got sudden attention with the discovery of Snowball, a sulfur-crested cockatoo that could synchronize head and body movements with the beat in several popular songs. Even though Snowball's behavior was only synchronized with the music for part of the time, he could adjust his movements to tempo changes of the songs (Patel et al., 2009a,b; Schachner et al., 2009). Parrots, such as Snowball, are vocal learners and vocal learning is associated with evolutionary modifications to the forebrain, which plays a key role in mediating a link between auditory input and motor output during learning (Petkov and Jarvis, 2012). As such linkage between auditory and motor areas in the brain is also required for beat induction (the ability to perceive a regular pulse in a varying rhythm, or real music; Honing, 2013) and audio-motor entrainment, Patel et al. (2009a,b) suggested that only vocal learning species might be able to show beat entrainment. A survey of YouTube movies searching for evidence of animal species that could entrain their behavior to music (Schachner et al., 2009) seemed to confirm this hypothesis: entrainment was only observed among those species that showed vocal learning, suggesting that vocal learning was a necessary, albeit not a sufficient, requirement for beat induction. However, further studies have shown the picture to be more complicated. Convincing evidence of entrainment with a musical beat has now also been established in a Californian sea lion, named Ronan (Cook et al., 2013). Although, sea lions belong to a clade of mammals (pinnipeds) that contains some vocal learners (Reichmuth and Casey, 2014), there is currently no evidence of vocal learning in this specific species, which potentially falsifies the generality of the hypothesis (Wilson and Cook, 2016). There is also some evidence of chimpanzees adapting their finger tapping to an external beat, although this seems limited to frequencies close to their spontaneous motor tempo (Hattori et al., 2013, 2015). Chimpanzees are considered vocal non-learners, although it can be argued that they show some vocal plasticity and adjustment (Watson et al., 2015), and hence that their limited abilities to synchronize match with their limited abilities for vocal learning. In addition, recent evidence for temporally coordinated rhythmic movements between a bonobo (also a vocal non-learning species) and a human drummer (Large and Gray, 2015) suggests that the link between vocal learning and beat induction may be less clear than initially anticipated. However, the most intriguing feature of the survey of Schachner et al. (2009) is that of those taxa that show vocal learning (for mammals: dolphins and whales, seals, bats, and elephants; for birds: parrots, songbirds, and hummingbirds—Janik and Slater, 1997; Petkov and Jarvis, 2012), evidence for beat induction was only present in several parrot species and elephants. With respect to the latter, the evidence that elephants are vocal learners originates from captive Asian elephants, which imitated truck sounds (Poole et al., 2005), and words of the caretaker (Stoeger et al., 2012). However, in this latter study, the speech sounds were produced by inserting the trunk into the mouth, i.e., in a way quite different from how elephants usually produce vocalizations. As it is possible to teach elephants to perform behavior patterns well outside their natural range by operant conditioning, it might well be that the speech imitations also arose by operant shaping of the vocalizations by the human caretaker, hence being based on a different mechanism from the auditory imitative vocal learning in other species. Taken together, this leaves the parrots as the only group showing both imitative vocal learning and beat induction. This calls for a re-examination of the link between vocal learning and beat perception and induction.

### Vocal learning and beat perception revisited: Are parrots special?

The YouTube survey (Schachner et al., 2009) shows a remarkable contrast between the parrots and other vocal learning birds (hummingbirds and songbirds). Seven different parrot species all show evidence of beat induction. Another parrot species that is not listed as observed to synchronize with music, the budgerigar, has since been shown to be able to peck a key in synchrony with a flashing light and a metronome, and could learn to adjust this pecking to some tempo changes (Hasegawa et al., 2011), although the adjustment to each new tempo was not spontaneous but was trained specifically. In striking contrast, the list of vocal learners contains 10 different songbird species and a hummingbird, none of which provided evidence for beat induction. In addition, three songbirds were erroneously classified under “vocal nonmimics” (nuthatch, bulbul, and babbler), with none of them showing beat induction. Thus, perhaps the question should be: why is it that various parrots, but no other vocal learning (or non-learning) birds, show beat induction? One possibility is that this difference is accidental. For instance, the total number of parrot movies is higher than that for the other bird species together, hence there may be a sampling bias. Or the difference might be related to behavioral differences between parrots and songbirds. Many parrot species show head bobbing or other body movements in their social interactions with conspecifics. If they are hand reared, as happens often with parrots, much of their social behavior will be directed to their human caretakers as a result of sexual or social imprinting (ten Cate and Vos, 1999) and a possible scenario might be that if their caretakers are dancing and moving on the beat, the parrots might be induced to do the same thing. Songbirds often lack such conspicuous rhythmic body movements in their natural behavior and may have less strong bonds with their caretakers as even captive ones are usually raised by their parents. Hence, they may possibly be less likely to provide evidence for beat detection and induction, even though they might be able to it. But it may also be that there is a more fundamental difference between parrots and other species. Showing beat induction requires at least two abilities: first the detection of a rhythmic pattern or beat in an external stimulus and next adjusting the frequency of some motor pattern to this input. Lack of beat induction may indicate lack of either or both of these abilities in other species. Thus, perhaps other bird species can detect rhythmic patterns in external stimuli, but lack the possibility to synchronize their behavior with it (see also Patel and Iversen, 2014).

Alternatively, non-parrot bird species might lack the ability for detecting rhythmic patterns in auditory stimuli altogether, due to the differences in brain pathways. It has been claimed that parrots have an enhanced vocal learning system, due to an extra song system that surrounds the song system shared with songbirds (Chakraborty et al., 2015). The motor pathway system that surrounds this shell song system shows gene expression profiles similar to the song system. These non-vocal motor brain regions are active during hopping and head bobbing movements (Feenders et al., 2008) and it is therefore suggested that this motor system is involved in entrainment (Chakraborty et al., 2015). Finally, the observed relationship between vocal learning and beat induction in parrots may be coincidental: parrots are vocal learners and show beat induction, but both may not be causally related or the relation may be due to some shared third factor underlying both.

To conclude: it is clear that it still is an open question why beat induction among birds has only been observed for parrots, calling for a further exploration of the topic. The observed contrast between parrots and songbirds make birds a particularly interesting group for comparative studies. Also, there are many vocal non-learning bird species, such as doves and pigeons, that show strong rhythmicity in their vocalizations and, finally, there are species not showing such rhythmicity. So comparing different species belonging to various avian groups may help to clarify the relation between vocal learning, beat and rhythm perception and beat induction. This may also reveal whether there is a categorical jump from synchronizing to pulses in natural behavior such as in flashing fireflies, to showing beat perception and synchronization, as implied by Patel et al. (2009a,b).

Recently, Arriaga et al. (2012) and Petkov and Jarvis (2012) proposed a vocal learning continuum hypothesis to accommodate the different levels of vocal learning ranging from vocal non-learners (like doves), limited vocal learners to complex-vocal learners (like parrots and songbirds). It may well be that there is also a more fine grained spectrum in rhythmic patterns (see also Ravignani et al., 2014), in particular when we shift the research focus from the production to the perception of rhythmic patterns. As the detection of rhythmic patterns, such as a pattern of repetitive identical inter-pulse-intervals or a higher order repetitive regularity in a rhythm, seems a first requirement for being able to move in synchrony with a beat, the central question in this paper concerns whether and which birds can detect such regularities in auditory patterns. And if so, is there a difference between species or groups of species in this ability? Or between vocal learners and non-learners?

### Can birds detect rhythmic patterns and regularity in auditory patterns?

In the first instance, asking whether birds, or any animal species, can detect regularity in an auditory pattern of, say, repeated pulses seems trivial. Studies on habituation have shown that various animals habituate more quickly to isochronous pulse series than to heterochronous ones (e.g., mice—Herry et al., 2007; zebra fish—Shafiei Sabet et al., 2015). However, this need not imply that they detect “isochrony” as such, as the distinction can be achieved by attending to local features, such as the differences in pause duration of one or a few inter-pulse intervals and by predicting the timing of a next event from the preceding interval, i.e., is based on a sensitivity to absolute, and not relative, timing (Honing and Merchant, 2014; Merchant and Honing, 2014; Merchant et al., 2015). Detecting isochrony as such, or rhythmic patterns more generally, involves a global process: detecting that events are regularly distributed over a longer series, irrespective of, for instance, the precise duration of the inter-pulse-intervals (see also Geiser et al., 2014). Rhythmic pattern detection thus concerns detecting a relational property. So, if an animal can distinguish between a regular, isochronous, pattern and an irregular, heterochronous one, a critical test to see whether this is based on having detected the global difference in regularity of the pattern is to see whether the discrimination is maintained after tempo transformations. So, what is known about such perceptual abilities in birds? To date, only a handful of bird species have been examined experimentally for their discrimination between isochronous and heterochronous sound patterns and/or for whether this discrimination is maintained with tempo changes of structured sound patterns. These are the domestic pigeon and several songbirds (starling, jackdaw, zebra finch). We briefly review these studies below.

#### Pigeons

Two studies examined whether pigeons could discriminate and generalize across different tempos. The first one (Farthing and Hearst, 1972) showed that pigeons subjected to a non-differential training in which they had to peck a response key to get food while being exposed to a regularly spaced train of pulses, generalized their response to slower, and faster pulse trains. This has also been demonstrated for quail chicks (Schneider and Lickliter, 2009), but it tells little on the ability to detect regularity or even tempo generalization in general, as the birds may have attended to the mere presence of any sound. Differential training, in which responses to one pulse rate but not to another one, were rewarded resulted in discrimination between the two rates and the differentiation was maintained with stimuli showing either higher or lower pulse rates than the training ones. However, this study did not examine the ability to discriminate regular from irregular rhythms.

A more recent study examined, among others, whether pigeons are able to detect and discriminate different meters (Hagmann and Cook, 2010). Using two sounds, different meters were constructed using the same pulse rate (180 bpm). The pigeons were able to discriminate the meters, but only if these differed substantially from each other (8/4 vs. 3/4). Further tests suggest that the pigeons might not have attended to the meter, but to the time difference between the beats. They did not transfer the discrimination to similarly structured stimuli consisting of other sounds, suggesting that their responses were also tied to the nature of the sounds. A second experiment on meter discrimination showed that the discrimination was maintained with faster tempos (200 and 220 bpm), but not with reduced tempos of the pulse (140 and 160 bpm). In a next experiment, the same birds were tested for their ability to discriminate an isochronous from an irregular pulse pattern. The pigeons did not succeed in this discrimination. Finally, it was examined whether they could discriminate between two different isochronous pulse rates (with pulse and pause durations scaled proportionally), similar to the study by Farthing and Hearst (1972). Three out of the four pigeons managed this discrimination and this was again generalized to slower and faster pulse trains. From these experiments, Hagmann and Cook (2010) conclude that, on the whole, pigeons were most likely attending to the intervals between pulses, rather than to the overall metric or regular structure of the sound strings.

#### Starlings

Starlings (songbirds) were tested for the perception of regularity and rhythm in a series of studies by Hulse S. H. et al. (1984), Hulse S. et al. (1984), Humpal and Cynx (1984). The birds were able to discriminate an isochronous (pulse duration 100 ms, intervals 100 ms) as well as a hierarchical pattern (four regularly spaced pulses followed by a longer pause and next followed by repetitions of this pattern) from a randomly generated heterochronous pattern with fluctuating pulse and pause durations. The discrimination was maintained with tempo changes in which pulse durations and intervals were extended or reduced proportionally (ranging from halving to doubling the tempo), although the strength of this discrimination was reduced for slower tempos (Hulse S. H. et al., 1984; Hulse S. et al., 1984). The discrimination was reduced when the inter-pulse-interval remained constant, and pulse durations varied, but not the other way around (Hulse S. H. et al., 1984; Hulse S. et al., 1984). The discrimination was also affected if pulse duration, but not the interval, was randomized or inverted, although it remained above chance level (Humpal and Cynx, 1984). Changing the pitch of the sounds affected the discrimination only slightly. Finally, the studies showed that starlings could discriminate two different rhythmic patterns consisting of four notes of different durations (50–50–300–300 ms vs. 50–300–300–50 ms), separated by longer pauses. Tempo transformation affected this discrimination, although it remained above chance in most cases (Hulse S. H. et al., 1984; Hulse S. et al., 1984). These experiments suggest that starlings are better than pigeons in attending to more global patterns of pulse trains, although most experiments show some loss of discrimination with various tempo transformations.

#### Jackdaws

A pioneering study on rhythmic perception in jackdaws (corvid songbirds) was done by Reinert (1965). He showed that a jackdaw could discriminate between two different auditory patterns with the structure ABAB and ABB respectively (A and B being different sounds). Among a series of other manipulations, he showed that the discrimination was maintained under tempo transformations (tempo training stimuli: 84 bpm; test stimuli: 66–192 bpm). A second jackdaw, trained to discriminate two other patterns, also maintained the discrimination with tempo transformations. Furthermore, the study demonstrated that the jackdaws maintained the discrimination between the patterns when the sounds making up the patterns were changed (varying timbre or pitch), suggesting that the jackdaws used relative and global, rather than local features like specific interval durations or tone characteristics, to distinguish the patterns. However, the jackdaws have not been tested with isochronous stimuli in different tempos, nor whether they could discriminate an isochronous from an irregular pattern. Also, the stimuli used in this study were always very short strings. So, although suggestive, conclusive evidence that jackdaws are really sensitive to an overall rhythm formed by a repeated pattern is still lacking.

#### Zebra finches

Zebra finches are a model songbird species for behavioral (e.g., Slater et al., 1988; Jones et al., 1996; Tchernichovski et al., 2001; Lipkind et al., 2013) and neural studies on song learning (e.g., Jarvis and Nottebohm, 1997; Haesler et al., 2004; Zeigler and Marler, 2008) as well as for comparative studies examining their abilities to discriminate various (speech) sounds or artificial grammar patterns (e.g., ten Cate, 2014). Also, a few studies examined their abilities for detecting or discriminating rhythm-like structures. Nagel et al. (2010) showed that zebra finches can distinguish the songs of two different males across various tempo transformations. Zebra finches can also detect prosodic patterns of edited speech sounds (Spierings and ten Cate, 2014) and can discriminate song elements arranged in an ABAB structure from an AABB structure (van Heijningen et al., 2009), and ABA structures from AAB structures (van Heijningen et al., 2013; Chen et al., 2015). Finally, exposure to a repeated series of regularly or irregularly spaced song elements induced differences in ZENK expression in two nuclei of the auditory system (NCM, CMM) (Lampen et al., 2014). These observations suggest that zebra finches might also be able to discriminate between different rhythmic patterns or between regular and irregular pulsed sounds and maintain this with tempo transformations. However, although zebra finches can discriminate a regular isochronous from an irregular stimulus, this discrimination was strongly reduced with tempo transformations (changing the inter-pulse-intervals, but not the pulse durations), even if the training consisted of several tempo variants of the isochronous and irregular stimuli (van der Aa et al., 2015). These data suggested that the zebra finches, like pigeons, attended strongly to specific local features of the individual stimuli, such as the exact duration of inter-pulse intervals, rather than the overall regularity of the stimuli. Whether they are able to use more overall features still remains to be demonstrated (see van der Aa et al., 2015, and below for a discussion).

To summarize the above overview: both pigeons and starlings are able to discriminate between two isochronous patterns in different tempos and maintain this discrimination with slower and faster tempos. However, this ability does not require perception of regularity as such, but can be achieved by attending to the duration of just one or a few intervals and generalizing from this to intervals that are more extreme to either one or the other end of the spectrum than the training ones. Although the currently available evidence is still limited, it suggests that all tested species can solve discrimination tasks when this can be done by attending to such local temporal features of the sounds, suggesting this is the “default” state birds use for auditory pattern recognition. This is also how pigeons discriminate (some) different metric patterns, generated by alternation of two types of sounds and how zebra finches discriminate regular from irregular pulse patterns (van der Aa et al., 2015). Starlings also attend to the durations of pulses and intervals when discriminating between isochronous and randomly spaced sounds varying in duration. However, their ability to maintain the discrimination over at least some tempo changes suggests that they might also be sensitive to the larger pattern. This may also be true for the jackdaws.

It can be concluded that the evidence that birds can attend to some more global “regularity” or “rhythmicity” as such, is still very limited. However, whatever evidence there is suggests that this ability may differ between species. The studies of Snowball, as well as some data of a gray parrot and the YouTube survey (Schachner et al., 2009) indicate that at least some parrot species have a quite well developed perceptual sensitivity for rhythm. The above review suggests the jackdaw as possible additional songbird candidate, but suggests also that this ability is poorer or even marginal in other songbird and non-songbird species. But, the experiments on all species are still equivocal on the issue, and more systematic comparative studies, focusing in particular on the discrimination between, and the responses to tempo transformations of regular vs. irregular stimuli are urgently required. Our experiments described below are meant to shed more light on such perceptual abilities.

### Can zebra finches and budgerigars perceive structural regularity?

In our experiments we compare a songbird species, the zebra finch, with a parrot species, the budgerigar, for their abilities to discriminate a regular, hierarchically structured stimulus from an irregular one. We chose the budgerigar because the study of Hasegawa et al. (2011) suggests that they are able to entrain their behavior to an audiovisual stimulus. Being a parrot species, we expect that they might also be able to attend to the more global features of temporal patterns in a perceptual discrimination task. For zebra finches, the current evidence for detecting pattern regularities is ambiguous: the study of van der Aa et al. (2015) suggest they attended only to local temporal features, but in the song discrimination study by Nagel et al. (2010) they were maintaining discrimination under tempo transformations. However, two songs changed in tempo might still be discriminable by other features that remained largely invariant after a tempo change, such as differences in the phonology of specific elements.

In the current study, we use training patterns that are similar in their (regular) inter-pulse-intervals, but differ in which pulses are accented, hence in their beat pattern. They thus show some hierarchical structure, providing the opportunity to examine how various local as well as more global temporal parameters affect the discrimination between the stimuli and whether this differs between the species. The birds are trained to discriminate between two hierarchical pulse strings, a regular one with one beat in each four pulses and an irregular string with the beats located at irregular positions. Subsequently, we test whether they generalize this discrimination to strings with modifications in the position and rate of the beat. This approach tests various hypotheses about which local and/or global features might be used when discriminating between regular and irregular strings.

## Materials and methods

### Subjects

Six male zebra finches and three female budgerigars were tested in this experiment. All zebra finches were between 120 and 321 days post hatching, the budgerigars were between 2 and 3 years old at the start of the experiment. The zebra finches were not subjected to previous experiments. The budgerigars had been used in a discrimination task with human speech and zebra finch sounds. Before the experiment, the animals were housed in group living facilities on a 13.5/10.5 L/D schedule and had food, water, and cuttlebone *ad libitum*. During the experiment, the L/D schedule was maintained, except for short dark periods as part of the experimental procedure. Water and cuttlebone were still *ad libitum*, the food availability was part of the experimental procedure and was monitored daily to ensure a sufficient level of food intake. The experiments were conducted in accordance to the animal experimentation guidelines of Leiden University. The protocol was approved by the Leiden committee for animal experiments, under DEC number 14071.

### Apparatus

All experiments were conducted in an operant conditioning cage [zebra finches: 70(l) × 30(d) × 45(h) cm, budgerigars: 70(l) × 60(d) × 60(h) cm]. Each operant cage was in a separate sound attenuated chamber and was illuminated by a fluorescent tube that emitted a daylight spectrum on a 13.5 L: 10.5 D schedule. A speaker (Vifa 10BGS119/8) was located 1 m above the center of the cage. The sound level was set to 70 dB at the location of the bird at the start of a trial (in front of sensor 1). The cage walls were made from wire mesh except for a plywood back wall which supported two pecking keys with LED lights. A food hatch was located in between these two keys, easily accessible to the birds. Pecking the left key (sensor 1) elicited a stimulus and illuminated the LED light of the key on the right (sensor 2). Depending on the sound, the bird had to peck sensor 2 or had to withhold its response. A correct pecking response resulted in access to food for 10 s. and an incorrect response led to 15 s. of darkness. Pecks during the sound presentation were not recorded as a response.

### Experimental design

#### Shaping

All birds started the experiment with a shaping procedure to get acquainted with the apparatus and the Go/No-go paradigm. This consisted of a 24-h acclimatization period with an opened food hatch, followed by a Go/No-go shaping procedure with one zebra finch song (Go sound) and one song element (No-go sound). Shaping lasted until the birds reached the standard discrimination ratio (response to Go sounds >75%, response to No-go sound <25%) for three consecutive days.

#### Discrimination training

**Figure 1:**
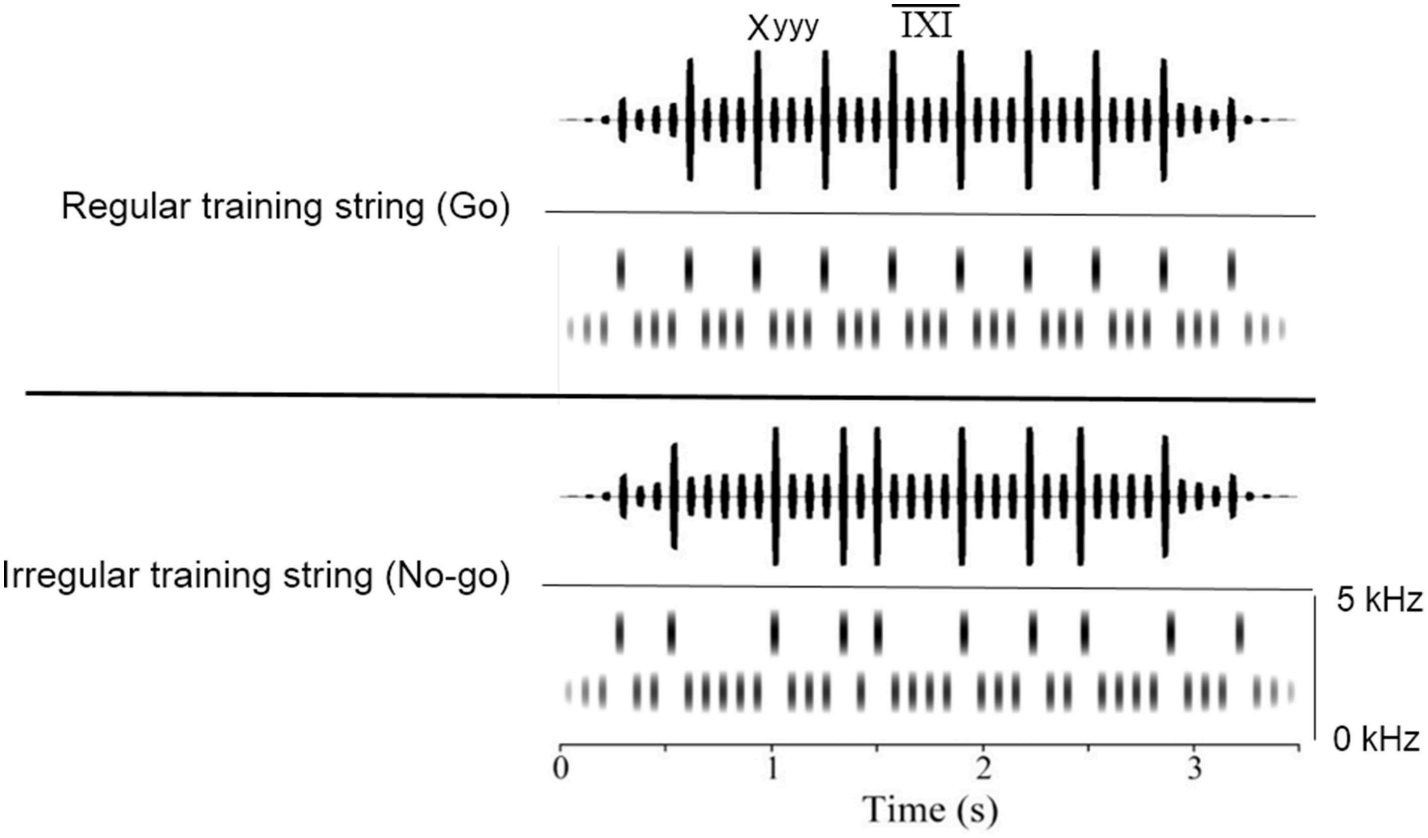
**Visualization of the regular and the irregular training strings**. The X-elements are indicated by the taller lines and the y-elements by the shorter lines. The IXI is the interval between two X-elements, measured from onset to onset. Note the fade-in and fade-out phases at beginning and end of the stimuli.

After shaping, all birds were trained to discriminate between one regular and one irregular string in the Go/No-go procedure (Figure 1). This training phase lasted until the bird reached the standard discrimination criterion for at least three consecutive days, after which it proceeded to the test phase.

#### Test phase

**Figure 2:**
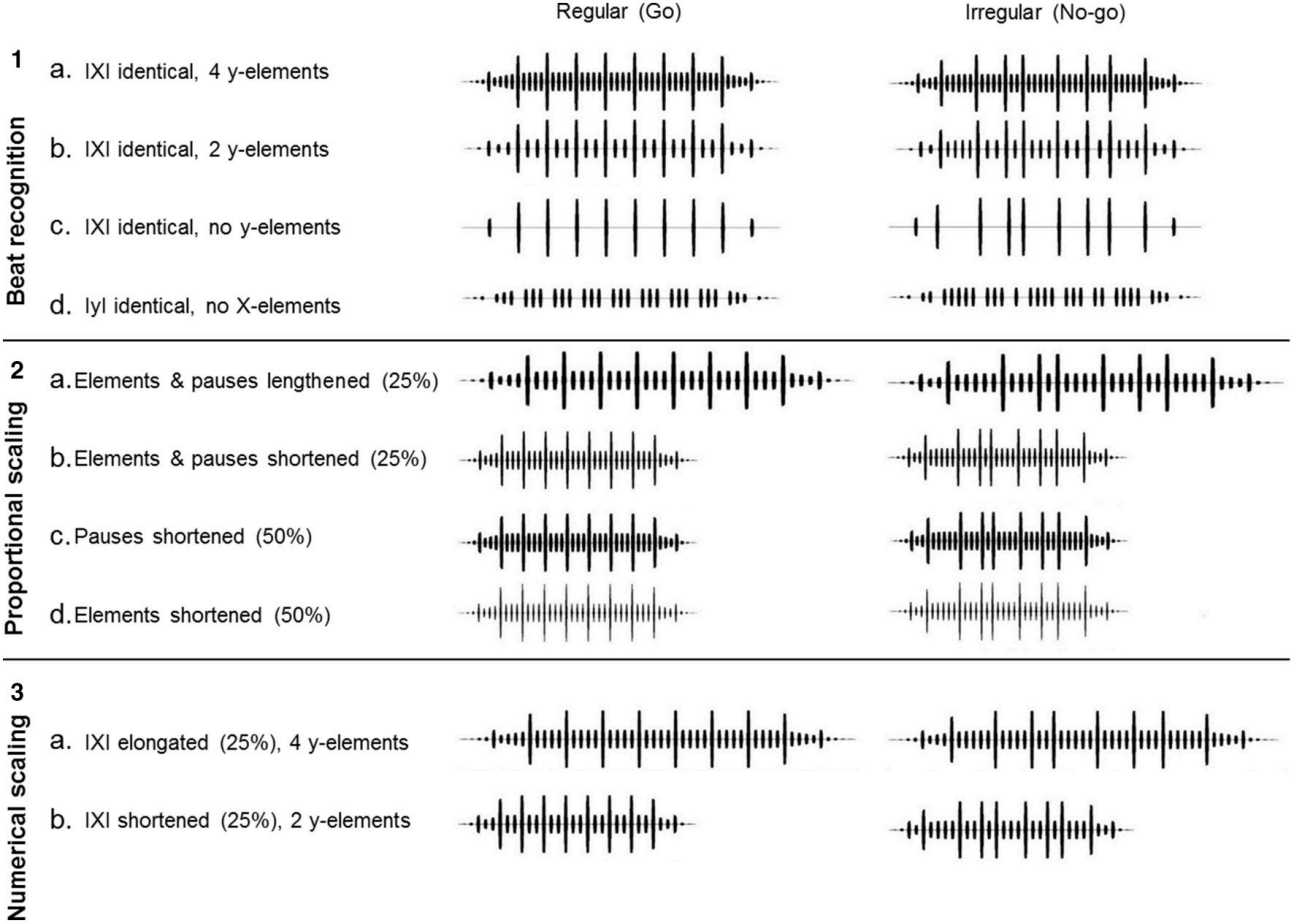
**Visualizations of the different test pairs with the regular and the irregular string variants, grouped in three test sets**. The longer vertical lines indicate the X-elements, shorter lines indicate the y-elements.

During the test phase, test strings were randomly played at 20% of the trials, whilst the other 80% of the trials remained training strings test strings (see Figure 2). Feedback in the form of food access and darkness was only given during training trials, never for the test trials. All test strings were presented randomly and the test phase lasted until each test string was presented 40 times.

#### Stimuli

Stimuli were strings consisting of two different tones, an (accented) X-element (4000 Hz, 40 ms and 80 dB) and a y-element (2500 Hz, 40 ms, and 70 dB, created in Praat version 5.4.01), separated by a short silent interval, the pause (40 ms). The elements were concatenated to form two hierarchically organized training strings: one regular (the Go sound) and one irregular (the No-go sound, Figure 1), each lasting 3.5 s in total. For both strings the interval between the elements was identical, what differed between them was the position of the X-elements, which affected the number of y-elements between two X-elements. The regular string was a concatenation of 10 equal units, where each unit consisted of one X-element, followed by three y-elements (Xyyy), all spaced equally. This concatenation created a stable 320 ms inter-X-interval (IXI), measured from onset to onset. The irregular string contained the same number of X- and y-elements as the regular string, but differed from this by variance in the number of y-elements between two X-elements. This variation ranged between one and five y-elements, creating a variation in the IXI between 160 and 480 ms. Both strings started with an additional three y-elements and had a fade-in and fade-out of 800 ms.

Test strings were created in a similar fashion as the training strings. They contained modifications in the duration and number of elements and pauses, thereby modifying the IXI and string length, while leaving the regular or irregular structure intact. Three main test sets were designed to systematically assess the effect of (1) modifications in the presence and location of elements, (2) the duration of elements and pauses, and (3) the number of elements on pattern detection (Figure 2, all regular strings used in this study are added as Supplementary Material).

##### Beat recognition: The role of number and presence of X- and y-elements

In these tests the IXI was kept identical to the training strings, whilst the number of y-elements in the string varied. If the birds discriminated the training strings by attending only to the duration of the accented pulses, i.e., the IXI interval, it is expected that varying the number of y-elements between the X's would not affect the discrimination. The test stimulus pair 1a (Figure 2.1) had an additional y-element between every two X-elements (four instead of three in the regular string). Test stimulus pairs 1b and 1c had a reduction of respectively one and three y-elements between two X-elements, creating regular strings with two and zero y-elements per IXI respectively. In the irregular strings each IXI is modified by adding or removing the same number of y-elements, with the limitation that there are never more than 5 or less than one y-element between two X-elements. Shortening or lengthening of the pause durations compensated for these modifications and kept the IXI identical to the training strings. An additional test (pair 1d) was ran with only y-elements and prolonged pauses to compensate for the lack of X-elements (Figure 2.1).

##### Proportional scaling: The role of element and pause duration and IXI

In this test the number of X- and y-elements was identical to the training stimuli (Figure 2.2). However, the IXI interval was varied because the stimuli for this experiment had modified durations of the elements and pauses, both shorter and longer than the elements in the training stimuli. The regularity and irregularity of the training strings stayed intact by equally modifying all elements or all pauses in a string. If the birds are attending to the regularity of always having four y-elements between the X-elements in the regular string, the discrimination should be maintained. Reduced responding would indicate that the zebra finches attend to finer temporal details of the stimuli. Two versions of this modification were created.

For stimulus pair 2a both the elements as well as the pauses were lengthened with 25%. Pair 2b had the elements and pauses shortened by 25% (Figure 2.2). The strings of pair 2c had the pauses shortened with 50%, but the elements stayed identical to those in the training strings. For pair 2d the elements were shortened with 50%, whilst the pauses stayed identical to the training strings. This reduced the IXI of pair 2c and 2d with 25%, similar to test pairs 2b (Figure 2.2).

##### Numerical scaling: The role of the number of y-elements and IXI

In this test the IXI's were extended and compressed to the same length as in test 2, by adding (pair 3a) or removing (pair 3b) one y-element per X-interval (Figure 2.3). This manipulation created strings identical in the numbers of y-elements between X-elements to test stimuli 1a and 1b, but in this case the duration of the elements and pauses remained identical to those of the training stimuli, creating strings with a smaller or larger IXI (Figure 2.3). This stimulus thus maintains the finer details of element and pause durations from the training strings and only moves the location of the X-element within the string.

An assumption underlying the training and test procedures and stimuli is that humans exposed to these stimuli would recognize the regularity of the stimuli without being explicitly told to do so, and that, after training, they classify all test stimuli appropriately. To validate this assumption, we trained a group of 24 adult human participants to discriminate between the training strings and tested them with test set 1 and 2. The participants convincingly discriminated the regular from the irregular strings of all test pairs (average response to regular stimulus = 0.88, average response to the irregular stimulus = 0.08, pairwise comparisons per test, all *p* < 0.01, see Supplementary Material). This indicates that, at least for humans, the regularity of the IXI intervals is recognizable, discriminable and generalizable. Thus far, we know that the sensitivity to temporal changes in birds, in the form of discriminating differences in duration or minimum integration time, is of a comparable level to that in humans (Dooling et al., 2000; Dooling, 2004).

#### Analyses

The response data of the zebra finches and budgerigars was recorded as binomial measurements (number of Go and No-go responses). For the analysis, these measurements were converted to fractions between 0 and 1, calculated as the cumulative Go responses toward the Go or No-go strings, divided by the total number of trials. For the zebra finches, these fractions were analyzed with a generalized linear model (glm) with test item (all test strings and the training go and no-go string) as fixed effect and the individual as the random measure. This gave a significant effect of the test item on the Go-fraction (*t* = 2.9, *p* = 0.004). Pairwise comparisons were made between the fractions of responses to the Go and the No-go string of each test set and between the responses to the training and test strings by using a Tukey's *post-hoc* test, corrected for multiple testing. All results shown in the Results Section originate from these *post-hoc* tests. Furthermore, we ran a glm on all individual data to analyze the response pattern of each zebra finch by using the binominal response measures to each of the 40 trials per test string per bird. Results of this glm showed a significant effect of test item on the test scores (*t* = 3.09, *p* < 0.001) and results shown further are from the pair-wise Tukey's *post-hoc* tests, restricted to only pairwise comparisons within each individual.

As only three budgerigars were tested, these data were only analyzed at the individual level. Like the zebra finch results, the responses of each budgerigar to each test string were measured as a binomial response. With a glm it was tested whether these scores differed over the test strings. The glm again showed a significant effect of test item on the test score (*t* = 2.76, *p* = 0.005). This was followed by a Tukey's *post-hoc* test with pairwise comparisons between the responses to the Go and No-go strings and between training and test strings within each individual. Results shown in the Result Section are from these *post-hoc* Tukey's test. All statistics were performed in Rstudio (version 0.98.1103).

## Results

### Training

The zebra finches required on average 10,245 trials to accurately discriminate between the regular and the irregular stimulus and complete the training. The three budgerigars learned the discrimination in 8495 trials on average.

#### Beat recognition

**Figure 3:**
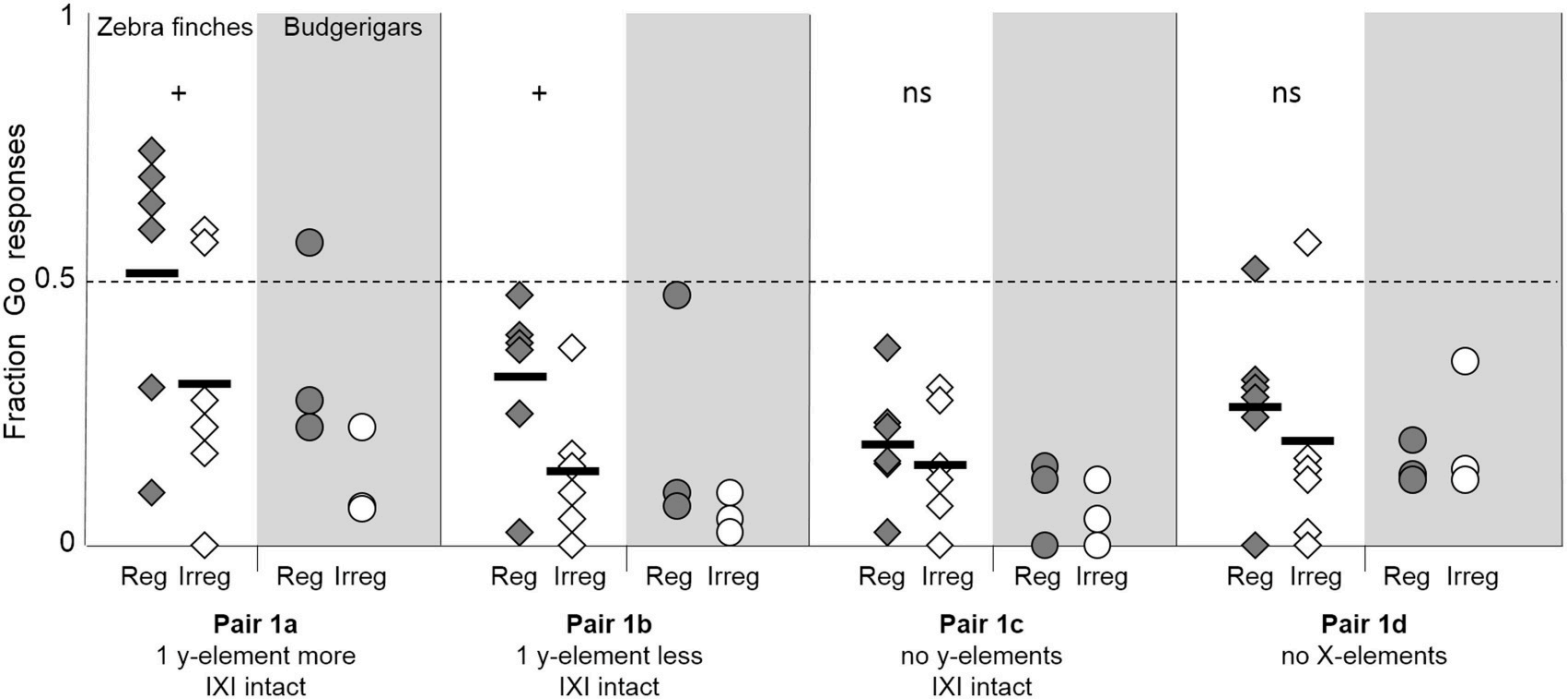
**Fraction of Go-responses toward the regular test strings (shaded diamonds) and the irregular test strings (open diamonds) for the four pairs of test set 1**. Horizontal bold lines shown the average fraction of Go responses of the six zebra finches. + symbols indicate a trend (0.05 < *p* < 0.10) toward a difference between the responses to the Go and to the No-go strings, ns indicates no significant difference (data for zebra finches only). Individual budgerigar results are shown with shaded circles (regular strings) and open circles (irregular strings). They were not tested at group level.

Maintaining IXI but varying the number and presence of y-elements reduced the discrimination between the regular and irregular test stimuli (Figure 3). Zebra finches showed a trend toward a discrimination between a regular and an irregular string when these strings had one additional y-element within each IXI compared to the training strings (4 y-elements, pair 1a − *z* = −5.08, *p* = 0.08). A discrimination bordering significance is shown when the strings have one y-element less in each IXI (2 y-elements, pair 1b) with elongated pauses (*z* = −3.37, *p* = 0.05).

Strings consisting of only X-elements with an identical IXI as the training strings (pair 1c) resulted in a reduction in the responses to the regular string, which was no longer discriminated from the irregular string (*z* = 0.02, *p* = 0.81). A similarly low number of responses and no discrimination was recorded when only the y-elements of the training strings were present in the test strings (pair 1d, *z* = −1.56, *p* = 0.22).

Two zebra finches (Z2 and Z5) correctly discriminated the strings with one y-element more in each IXI (pair 1a: Z2: *z* = −3.98, *p* < 0.01; Z5: *z* = −4.65, *p* < 0.01), whilst the other four zebra finches did not discriminate (all *z* > −2.13, *p* > 0.25). None of the individuals discriminated between the regular and irregular string of pair 1b—one y-element less per IXI, 1c—no y-elements, and 1d—no X-elements (all *z* > −1.33, *p* > 0.5).

The budgerigars also showed reduced responses to test stimuli with an identical IXI to training, but with modified numbers of y-elements (Figure 3). Nevertheless, one budgerigar (B1) correctly discriminated the regular and irregular strings both with 4 y-elements (pair 1a) and 2 y-elements (pair 1b) between the X-elements (pair 1a: *z* = −7.23, *p* < 0.01; pair 1b: *z* = −6.14, *p* < 0.01). The other two budgerigars (B3 & B2) did not discriminate these strings (pair 1a and pair 1b: both z > 0.8, *p* > 0.9).

Similarly to the zebra finches, budgerigars did not discriminate between the regular and irregular string when only the X-elements (pair 1c) or only the y-elements (pair 1d) were present (pair 1c: all z > 0.3, *p* > 0.9; pair 1d: all *z* > 0.04, *p* > 0.9).

These results show that discrimination between regular and irregular strings was only partially maintained when the IXI remained constant whilst the number of y-elements varied. Only two zebra finches and one budgerigar discriminated between strings with one extra y-element in the IXI. None of the birds maintained the discrimination when only the y-elements and their intervals were present. It is clear that both element types were required and that whatever the birds might have used to discriminate the training strings, it was not just regularity, nor exact duration of the IXI.

#### Proportional scaling

**Figure 4:**
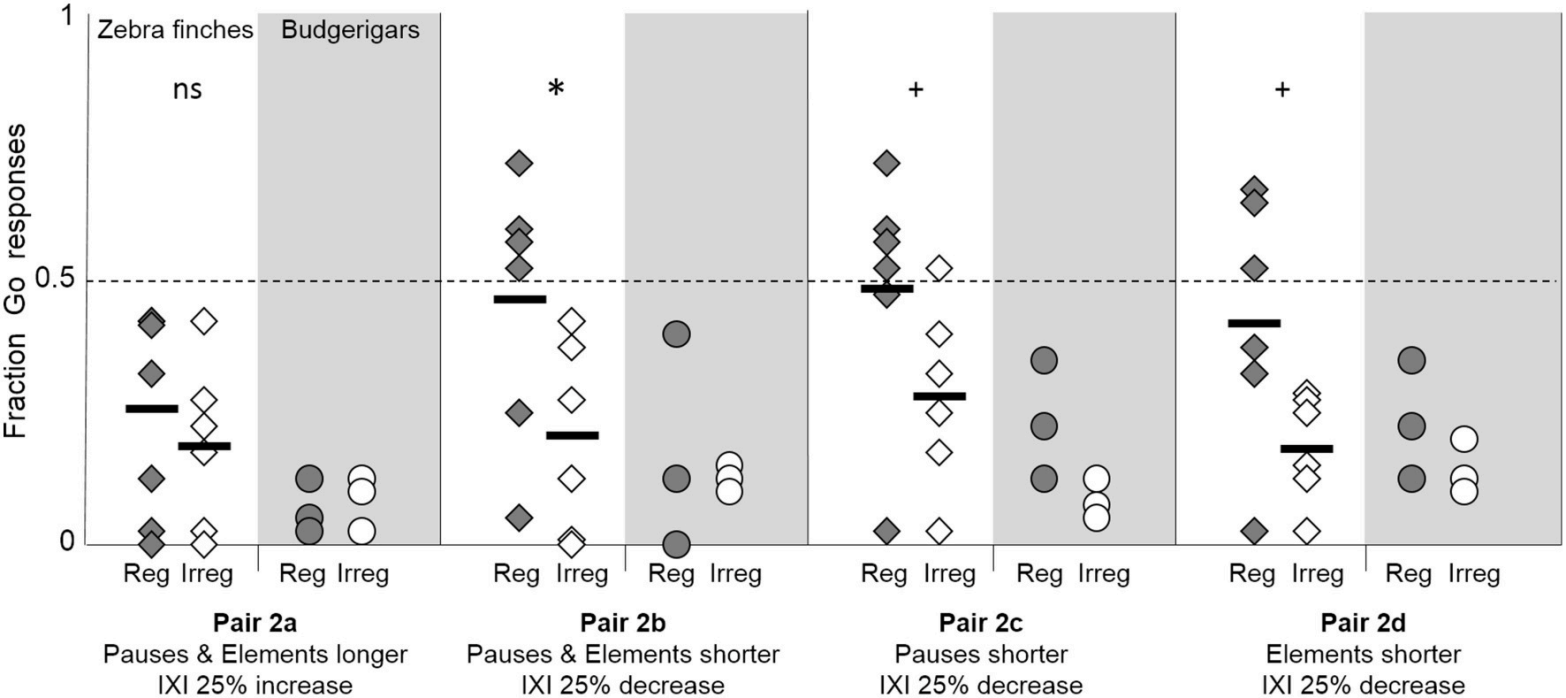
**Fraction of Go-responses toward the regular strings (shaded diamonds) and irregular strings (open diamonds) with elongated (2a) or shortened (2b) elements and pauses, as well as only shortened pauses (2c) and only shortened elements (2d) creating a 25% increase or decrease of the IXI**. Black lines shown the average fraction of Go responses of the six zebra finches. Asterisks indicate a significant difference between the responses to the Go and to the No-go strings, + symbols indicate a trend, ns indicates no significant difference. Individual budgerigar results are shown with shaded circles (regular strings) and open circles (irregular strings).

Modifications in the duration of both pauses and elements evoked different effects depending on the direction of the modification (Figure 4). Zebra finches showed no discrimination between the regular and irregular strings when both elements and pauses were elongated by 25% (pair 2a: *z* = −1.73, *p* = 0.14). However, they did make a correct discrimination between the regular and irregular string when both elements and pauses were shortened by 25% (pair 2b: *z* = −6.31, *p* = 0.03).

Keeping the element duration identical to training stimuli but shortening the pauses in the strings by 50% (pair 2c), showed a bordering significant result toward good discrimination by the zebra finches (*z* = −3.98, *p* = 0.05). A similar trend in the responses was found when the elements were shortened, but pauses kept similar to the training stimuli (pair 2d: *z* = 3.29, *p* = 0.06).

Two zebra finches (Z2 and Z5) discriminated the regular and irregular string with shorter pauses and elements (pair 2b: Z2, *z* = −6.86, *p* < 0.01; Z5, *z* = −6.92, *p* < 0.01, Figure 4). These were the same individuals that discriminated the regular and irregular string from pair 1a (4 y-elements per IXI). One zebra finch made a correct discrimination when only the pauses were shortened (pair 2c, Z5, *z* = −5.47, *p* < 0.01). Two other zebra finches correctly discriminated when the elements were shortened (pair 2d, Z2 and Z3, *z* = −4.98, *z* = −4.09, both *p* < 0.01).

Budgerigars hardly responded to strings with modified pause and element durations (Figure 4). Irrespective of the type of modification and whether the elements, the pauses, or both were modified, none of the budgerigars discriminated between the regular and the irregular strings (pair 2a: all *z* > 0.03, *p* > 0.9; pair 2b: all *z* > 0.07, *p* > 0.6; pair 2c: all *z* > −1.4, *p* > 0.2; pair 2d: all *z* > −2.8, *p* > 0.4).

The duration of the elements and pauses influenced the birds' discrimination abilities differently in the two species. While budgerigars failed to discriminate between proportionally scaled strings, zebra finches' discrimination was maintained with shortened elements and pauses, although it was lost for all individuals when elements and pauses were elongated. There was no clear indication that reductions of elements, of pauses, or of both differ in their effect. The results suggest that the zebra finches showed at least some generalization of the discrimination when the number of y-elements between X-elements is left intact.

#### Numerical scaling

**Figure 5:**
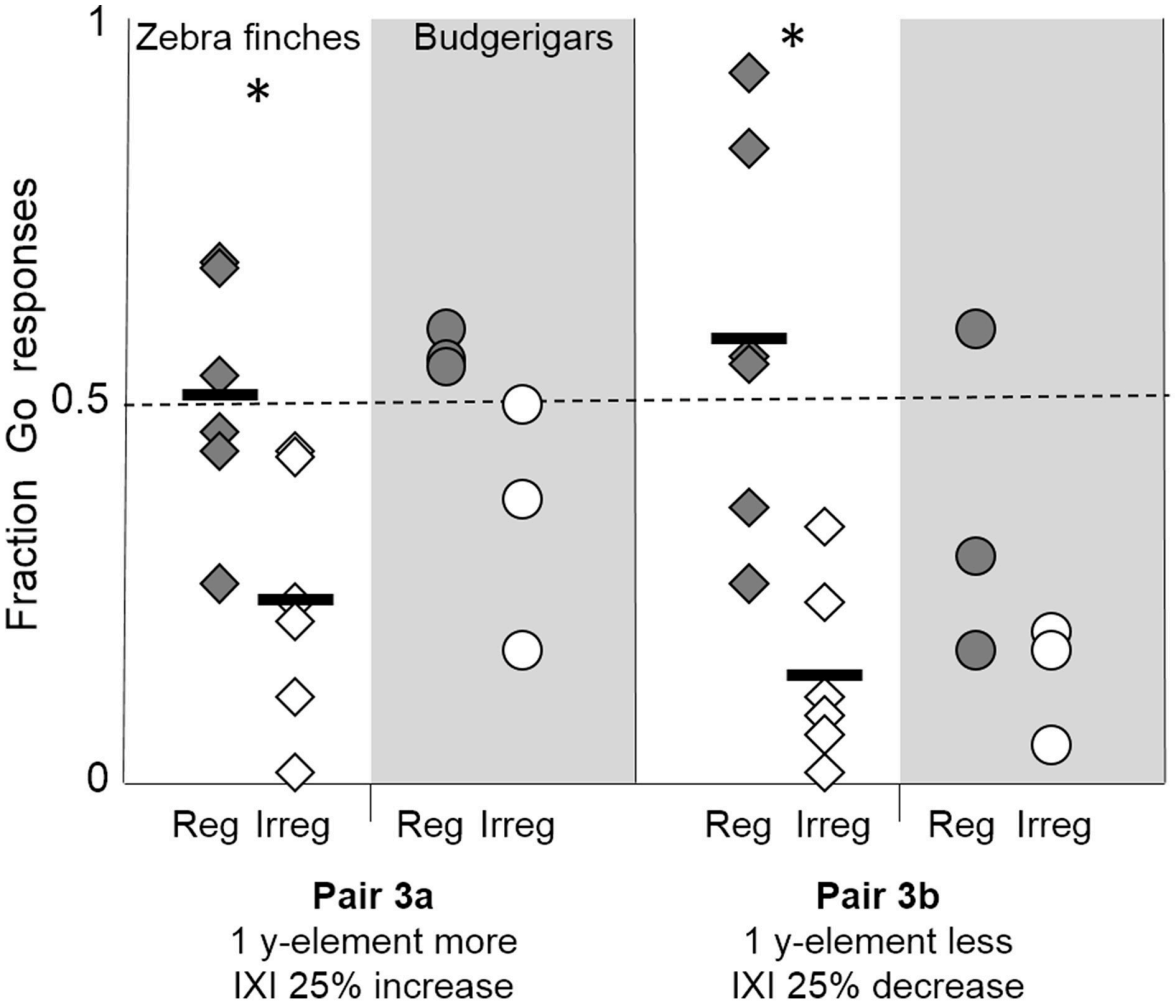
**Fraction of Go-responses toward the regular strings (shaded diamonds) and irregular strings (open diamonds) with one y-element more (4 y-elements, pair 3a) or less (2 y-elements, pair 3b) compared to the training strings, creating a 25% increase or decrease of the IXI**. Black lines shown the average fraction of Go responses of the six zebra finches. Asterisks indicate a significant difference between the responses to the Go and to the No-go strings. Individual budgerigar results are shown with shaded circles (regular strings) and open circles (irregular strings).

The zebra finches maintained their discrimination between regular and irregular pulse strings when each IXI contained 4 y-elements, one y-element more than in the training strings, and thus had a 25% increase in duration of the IXI compared to training (pair 3a, Figure 5). Overall, zebra finches showed more Go-responses to the regular string than to the irregular string (*z* = −9.88, *p* < 0.001). The same discrimination ability was found when there were 2 y-elements in each IXI, creating a decrease in duration of the IXI by 25% (pair 3b: *z* = −6.61, *p* = 0.002). The level of discrimination between the regular and irregular string did not differ between these two manipulations (*z* = 0.06, *p* = 0.45).

All but one zebra finch discriminated between the strings with 2 y-elements and a shorter IXI (pair 3b, Z1, Z2, Z3, Z4, and Z5). Also, two zebra finches made this discrimination when there were 4 y-elements and the IXI was elongated (pair 3a: Z1, *z* = −6.23, *p* < 0.01, and Z4, *z* = −8.45, *p* < 0.01).

One budgerigar (B1) discriminated correctly when each IXI contained an extra y-element, creating an IXI increase of 25% (*z* = −5.45, *p* < 0.01), whilst the other two budgerigars did not discriminate these strings (both *z* > −2.24, *p* > 0.7, Figure 5). When the IXI was reduced by 25% by removing 1 y-element between the X-elements, again one budgerigar (B2) made a correct discrimination (*z* = −4.33, *p* < 0.01), while the other two budgerigars did not discriminate (both *z* > 2.17, *p* > 0.15).

These results confirm that the IXI did not need to be identical to the training strings for the birds to correctly discriminate between a regular and an irregular pulse string. In these test strings, the durations of the elements and pauses were maintained, but the number of y-elements varied. This also demonstrates that in this test the birds did not use the exact number of y-elements between two X-elements, nor the location of the X-element to discriminate between the training strings. Rather it seems that generalization to longer and shorter regular patterns was at its best if the element and pause durations were kept identical to the training stimuli.

##### Comparing responses to training and test strings

A comparison between the responses of the zebra finches to the training and to the test strings revealed that although there were differences in the responses toward regular and irregular strings in the various tests, the average fraction of Go responses to the regular test strings was always lower than the responses to the regular training strings (pairwise comparisons regular test strings ~ regular training string, all *z* < −9.92, *p* < 0.01). Nevertheless, the zebra finches always responded more often to the regular test strings than they did to the irregular training strings (pairwise comparison regular test strings ~ irregular training, all *z* < −4.54, *p* < 0.01). The irregular test string of pair 1a (increased number of y-elements, identical IXI), pair 2c (pauses shortened by 50%), and pair 3a (IXI elongated by extra y-element) were the only stimuli to which the birds responded more often with a Go response than they did to the irregular training string (pairwise comparisons irregular test strings ~ irregular training string, pair 1a, pair 2c and pair 3a: *z* < −5.68, *p* < 0.01, all other *z* > −1.7, *p* > 0.1).

The budgerigars also responded less to all regular and irregular test strings than they did to the regular training strings (all *z* < −5.78, *p* < 0.01). However, some regular test strings got more Go responses than the irregular training strings. When one y-element was added between two X-elements and the IXI was increased correspondingly (pair 3a), all budgies responded with more Go responses to the regular string than to the irregular training string (all *z* < −6.38, *p* < 0.01). Additionally, one budgerigar (B2) also responded more strongly to regular test strings than to irregular training strings when there was one y-element removed (pair 3b), when there were no y-elements (1c) or when both pauses and elements were elongated (2a) (all *z* < −3.73, *p* < 0.02). Budgerigar B3 responded stronger to the regular test string than to the No-Go training string when one y-element was added or removed, but the IXI stayed identical to training (pairs 1a and 1b, *z* = −5.78, *z* = −5.21, both *p* < 0.01).

## Discussion

Zebra finches and budgerigars can learn to discriminate between regular and irregular pulse strings in a Go/No-go operant training procedure. If the birds, like humans (see Supplementary Material), would make the discrimination based on differentiating on the basis of presence or absence of regularity, one would expect that all regular test stimuli would obtain similar Go-scores to the regular training stimulus, and be preferred consistently over the irregular test stimuli. This was not the case. Responding was considerably lower to regular test stimuli than to regular training stimuli and there is no consistent preference for the regular over the irregular test stimulus. However, several regular test stimuli got more responses than their irregular counterpart. So, what might underlie the differential responding?

Our three test-sets (see Figure 2) provide insights into the features of the regular and irregular training strings that zebra finches and budgerigars used when discriminating between them. The first test set showed that the birds did not discriminate the regular and irregular strings by attending exclusively to the IXI, nor by attending to the pattern of the y-elements. Apparently both element types are required to make the discrimination. However, some individuals maintained the discrimination with an increased number of y-elements and constant IXI. Test set two revealed that the zebra finches, but not the budgerigars, tended to maintain discrimination between the regular and irregular strings if the number of y-elements remained constant, but duration of pauses and/or elements were shortened. Discrimination was absent in both species when both elements and pauses were longer than the training strings. Finally, the third set showed that both zebra finches and budgerigars can discriminate between regular and irregular strings in which the number of y-elements and IXI is varied, provided that the duration of elements and pauses is maintained.

Concentrating on the statistically significant findings of the different individuals shows the presence of three main patterns. (1) Memorization without generalization: one budgerigar (B3) and one zebra finch (Z6) did not discriminate between any of the test string sets, suggesting that they memorized the training strings providing a food reward and discarded all deviating strings. (2) Generalization across varying IXI when local features of the test strings, like element and pause length, were identical to the training strings. One budgerigar (B2) and three zebra finches (Z1, Z3, and Z4) discriminated strings with more or fewer y-elements between the X-elements and therefore a longer or shorter IXI (pair 3a and 3b). (3) Generalization with local variation: One budgerigar (B1) and two zebra finches (Z2 and Z5) discriminated strings with longer or shorter elements and IXI's, indicating that they were able to generalize regularity beyond local features. However, each individual had a specific subset of test strings which it discriminated, showing that there are still some specific local features that played a role during discrimination.

The individual variation among zebra finches, ranging from a focus on the exact structure of the stimuli to one with additional attending to a more global structure, has also been found in experiments in which zebra finches had to distinguish among string sets based on different artificial grammar patterns (van Heijningen et al., 2009, 2013; Chen et al., 2015) and may hence reflect a variation in more general cognitive abilities. Our current results are also in line with the suggestion arising from reviewing earlier studies (see Section Introduction) that birds have a primary strategy to pay attention to local temporal features, in this case the duration of the elements and the pauses between them, for auditory pattern recognition. However, also in the present study it is clear that this initial strategy might be accompanied by a sensitivity to more global features, like the regularity of the pulse strings, as is shown by the correct discrimination between strings in which the IXI is modified by adding y-elements, but keeping identical element and pause durations (see pair 3a in Figure 5). Some sensitivity to regularity is also suggested by the finding that zebra finches responded more to the regular test strings than to the irregular training strings. The differentiation among the test stimuli of each type also suggests that they most likely based their responses on comparing test strings with both the regular as well as the irregular training string. In our experiment we used only a single regular and a single irregular training string. While this was sufficient for humans to classify novel strings as being regular or irregular this was not the case for the birds. However, it may be that if the birds had been trained on a set of regular and irregular stimuli they might have shifted more clearly from using local features to using the global feature of regularity.

Our zebra finch results seem somewhat in between those obtained by Nagel et al. (2010) and those of van der Aa et al. (2015) for the discrimination between two stimuli of which the temporal parameters were varied compared to the training stimuli. The study by Nagel et al. (2010), using songs from two different males, showed that discrimination of manipulated stimuli was similar to those of the training stimuli with changes in song duration of even >25%.(van der Aa et al., 2015) used one type of pulse, separated by isochronous or heterochronous intervals and showed that discrimination between regular and irregular test stimuli disappeared with a 25% tempo change. Our stimuli were more complex than those of van der Aa et al. by using two types of elements, but lacked the phonological features present in full songs. In the present study, a 25% tempo change did affect some, but not all of the discriminations. Zebra finch songs differ in many features, such as the pitch contours, element length, amplitude modulations and formant patterns. Some of these features might have remained recognizable in the study of Nagel et al. (2010) where the songs were proportionally scaled, allowing the zebra finches to use these features, instead of the rhythmic ones. Hence we cannot be sure that the rhythmic structure of the songs was used in maintaining the discrimination in that study. The results of van der Aa et al. suggested that zebra finches attend in particular to local features, in that case the exact duration of inter-onset intervals. Our current results support this partly, as discrimination seems most affected when durations of pauses and elements were manipulated, but also show that some discrimination was maintained with a shortening, but not with a lengthening of element and pause durations. Maintenance of some discrimination between regular and irregular stimuli with proportional scaling of both pauses and elements has also been shown for starlings (Hulse S. H. et al., 1984; Hulse S. et al., 1984) and pigeons (Farthing and Hearst, 1972). It is of interest that for both of these species a decrease in tempo resulted in a stronger reduction of discrimination than an increase, similar to what is observed in the current experiment. The starlings appeared more sensitive to changes in tone length than changes in inter-onset interval, while the zebra finches in our study seemed to give equal weight to both.

The reduction in discrimination resulting from proportional scaling was, for both zebra finches and budgerigars, stronger than that for starlings, which maintained good discrimination with a 40% tempo change (Hulse S. et al., 1984). Hulse S. et al. (1984) interpreted their findings as indicating at least some sensitivity to rhythmicity for starlings. Our results are less conclusive on this issue. They suggest that both zebra finches and budgerigars showed some sensitivity to stimulus regularity, but only when some local features remained invariant. Similar ambiguous findings were observed in other studies of rhythm perception in birds, as discussed in the introduction. For example, pigeons could discriminate between meters with different pulse rates and between different regular pulse strings, but not between a regular and an irregular pulse string (Hagmann and Cook, 2010). Furthermore, they could not generalize the meter discrimination to pulse strings with similar rhythmic features, but different sound items. These results indicate that the discrimination by the pigeons was based on local phonological and temporal features, such as the absolute inter-pulse-intervals, and not on the global regularity of the strings. In a follow-up experiment using some of the same birds as used in the current experiment (Spierings et al., unpublished), we also found that both species hardly responded when X- and y-elements were replaced by elements of the same duration but differing in phonetic structure. In contrast to the pigeons, starlings (and possibly jackdaws—(Reinert, 1965), see Section Introduction) were able to discriminate between regular and random pulse strings, and could generalize this discrimination to some modifications of these strings, indicating that they might have attended to the global rhythmic feature of the pulse strings (Hulse S. H. et al., 1984; Hulse S. et al., 1984; Humpal and Cynx, 1984). However, just like the zebra finches, the starlings also discriminated best if some local features remained identical to the training strings, such as pulse duration, whilst changes in others such as the pitch of the pulses, did not affect the discrimination.

Abilities related to auditory-motor rhythm production have so far mainly been shown in avian species belonging to the parrot clade. Not only larger parrots can, to a certain extent, synchronize their body movements with a beat, but also the smaller budgerigars have shown rhythmic entrainment (Hasegawa et al., 2011). Nevertheless, this particular experiment might not have required regularity perception from the budgerigars. They were required to peck on a key at certain regular interval, indicated by a light and a sound. Repeating the previously heard or seen interval, i.e., attending to absolute timing, might have allowed the birds to correctly entrain to the presented rhythm. The budgerigars in the present study did not use the general regularity of the strings to discriminate, since they only discriminated between specific regular and irregular strings.

So, both zebra finches and budgerigars were in general not using the global regularity when discriminating these strings, but both could attend to some aspects of regularity. This is in contrast to another study, in which budgerigars and zebra finches were tested on their rule learning strategies (Spierings and ten Cate, in revision). That study showed that zebra finches used local, positional information to discriminate song element triplets (XYX and XXY), whilst budgerigars used a global strategy and attended to the structure of the strings. This resulted in a generalization of the structural rules by the budgerigars, but not by the zebra finches. One noticeable difference between that study and the current on is that Spierings and ten Cate (in revision) used a set of exemplars of the XYX and the XXY string during training, whereas in the current study the animals were trained with one regular and one irregular string. Less variation in training strings might have reduced the attention given to the general regularity-irregularity difference. Nevertheless, if the difference in regularity of the strings was the most prominent strategy to discriminate them, this strategy should also be employed with only one exemplar of each, as shown by the human subjects (see Supplementary Material).

One way of interpreting the existing literature and the current results is to distinguish between at least three types of perceptual biases that might characterize time and rhythm perception in birds and other animals. These three types are a bias for local features of auditory elements (such as pitch, amplitude, duration), a bias for more global prosodic features (such as pitch contour or amplitude contour), or a bias for the temporal structure, such as inter-beat-intervals. In the current study, most individuals seem to use local temporal features as their primary strategy in solving the discrimination task. We refer to this as the local feature bias hypothesis. This hypothesis suggests a preference in birds for local elements (such as duration, inter-onset interval, pitch, amplitude, or timbre) in perception and discrimination tasks and a lower sensitivity to whether they are part of a more global temporal structure, be it isochronous, heterochronous or metrical. This is not to say that zebra finches and budgerigars cannot take advantage of the global structure; it is just not their preferred strategy in solving this type of discrimination tasks.

To summarize the results of the current experiment and those reviewed in the introduction of our study: there is between and within species variation in how well different birds are able to detect regularity of pulse strings. However, while the vocal non-learning pigeons seem to perform poorest on this, there is only a gradual difference with vocal learners such as zebra finches and budgerigars, which in turn show a gradual difference with starlings and jackdaws. Also, if there is, as our review suggested, a difference between parrots and other bird species in sensitivity to regularity and rhythm, it does not hold for the budgerigar. Also, the currently available data show no systematic differences among vocal learners and non-learners. So, we suggest, similar to what Merchant and Honing (2014) suggested for primates, that the current data show a continuum (instead of a categorical jump) in the ability to detect regularity and rhythmicity. This idea is similar to the continuum hypothesis suggested for vocal learning by Arriaga et al. (2012) and Petkov and Jarvis (2012). However, it should be realized that the number of species tested for their abilities to perceive regularity or rhythm is still limited and the test methods and stimuli varied. Hence, there is a need to extend experiments to other avian groups, both vocal non-learners as well as some vocal learning groups that are considered to be more advanced in their cognitive abilities (such as large parrots and corvids) and therefore may be expected to have more elaborate rhythm perception.

## Author contributions

CtC, MS, JH, and HH designed research; MS and JH performed research; MS and JH analyzed data; CtC and MS wrote the paper and JH and HH improved the paper.

### Conflict of interest statement

The authors declare that the research was conducted in the absence of any commercial or financial relationships that could be construed as a potential conflict of interest.
