# Supplementary material for: Can Birds Perceive Rhythmic Patterns? A Review and Experiments on a Songbird and a Parrot Species
Source: Front Psychol. 2016 May 19;7:730. doi: 10.3389/fpsyg.2016.00730 (PMC4872036; doi:10.3389/fpsyg.2016.00730)
Supplement: Supplementary file 1 [file DataSheet1.docx]

Supplementary information

**Can birds perceive rhythmic patterns? A review and experiments on a songbird and a parrot species.**

Carel ten Cate ^1^*, Michelle Spierings ^1^*, Jeroen Hubert*, Henkjan Honing#

* Behavioural Biology, Institute of Biology Leiden & Leiden Institute for Brain and Cognition, Leiden University, Leiden, Netherlands

# Amsterdam Brain and Cognition, Institute for Logic Language and Computation, University of Amsterdam, Amsterdam, Netherlands

^1^ These authors contributed equally

To verify that humans treat our stimuli by attending to their regularity, adult human participants were trained and tested in a Go/no-go paradigm on a computer. The participants were instructed on the functioning of the go/no-go, and were subsequently trained and tested with the same strings as were used for the birds.

Methods

Subjects

Twenty-four human participants (age 29.5 +/- 2.7) were trained and tested in this experiment, one extra participant was unable to successfully finish the training. All participants were native Dutch speakers, reported to have no hearing problems..

Apparatus and experimental design

The participants were asked to sit in front of a computer and received a set of headphones (Denon DJ DN-HP1000) with a sound level of 70dB. The Go/No-go paradigm was written in Processing, a script based design program. Before the start of the experiment participants were instructed to click at a speaker icon that appeared in the middle of the screen. This click started a trial and the playback of a stimulus, followed by the appearance of a blue rectangle. The participants were instructed to either click or not click on this rectangle before it would disappear after 3.5 seconds. Feedback was provided with a happy smiley and a “ping” sound after a correct Go response and a sad smiley and a loud low sound after an incorrect Go response.

After the instruction, the participant started the training. Participants had to learn to discriminate between a regular string (Go sound) and an irregular string (No-go sound, figure 1). After they reached the standard discrimination ratio, more than 75% correct Go responses and less than 25% incorrect No-Go responses within a block of 30 trials, they continued to the test phase.

Participants received a written instruction on the screen notifying that they continued to the test phase and would not receive any feedback. Test items were presented in blocks of 20 trials, 16 test strings and 2 repetition of the 2 training strings. The test was finished when a participant finished 4 blocks of 20 trials.

Stimuli

The training stimuli were identical to those used for the zebra finches and budgerigars (figure 1). Also the same test stimuli were used, although humans were only tested on test set 1 and 2 (figure 2.1 and 2.2). This means they received test strings with one more y-element between two X-elements, but with an identical IXI (inter-X-interval) to the training (pair 1a). Strings with one y-element less between two X intervals, with an identical IXI (pair 1b). Strings with no y-elements between two X-interval, again the IXI stayed identical to training (pair 1c). And strings without X-elements, where the y-elements remained in identical positions compared to the training string (pair 1d).

In test set 2 the IXI were longer or shorter than the training strings. The participants heard strings with longer elements and pauses, creating an IXI increase of 25% (pair 2a) and strings with shorter elements and pauses, resulting in a decrease in the IXI of 25% (pair 2b). Furthermore, they heard a strings with only the pauses shortened, creating an IXI decrease of 25% (pair 2c), and a string with only the elements shortened to create an IXI decrease of 25% (pair 2d).

Analyses

The Go and No-go responses of the participants were calculated as fractions correct and fraction incorrect by the following formula’s: Fraction correct = correct Go/ (correct GO + incorrect Go), fraction incorrect = incorrect Go/ (correct Go + incorrect Go). These fractions were then analyzed with generalized linear model and Tukey’s post-hoc test in which the correct and incorrect fractions per test were pitted against each other.

Results

24 out of 25 participants learned to discriminate between the regular and the irregular training string, they needed on average 1.7 blocks of 30 trials. One participant did not learn the discrimination within 120 training trials and did not proceed to the test.

Regardless of the test pair, humans discriminated between the regular and the irregular strings (figure S1, all *p*<0.01). However, the discrimination between the regular and the irregular string without X-elements (pair 1d), was less strong compared to the other tests (all comparisons with pair 1d *p*<0.05). No difference in discrimination strength was found between the other test pairs (all *p*>0.1).

Sound files

The wave files of the training stimuli and the regular test stimuli used for both the bird and human (only test 1 and 2) studies are added as supplementary files. The filenames correspond to the stimuli as follows:

| Audio file number | Stimulus |
| --- | --- |
| 1 | Regular training string |
| 2 | Irregular training string |
| 3 | Test 1a Regular |
| 4 | Test 1b Regular |
| 5 | Test 1c Regular |
| 6 | Test 1d Regular |
| 7 | Test 2a Regular |
| 8 | Test 2b Regular |
| 9 | Test 2c Regular |
| 10 | Test 2d Regular |
| 11 | Test 3a Regular |
| 12 | Test 3b Regular |


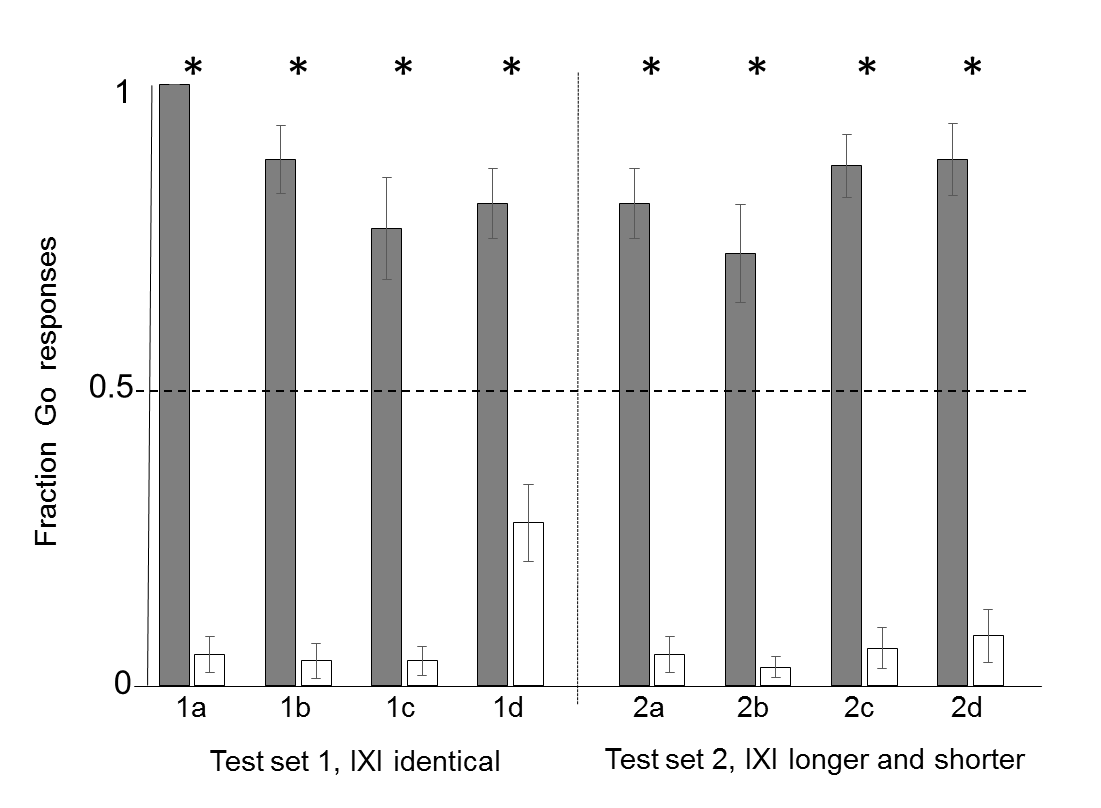


Figure S1. Responses of human participants to the different test pairs. The shaded bar shows the fraction of correct responses to regular (Go) strings, the open bar shows the fraction of incorrect Go responses to irregular (No-go) strings. Bars show the average score, whiskers the standard error of the mean. Asterisks indicate a significant difference obtained in Tukey’s post-hoc tests from a GLM.
